# Rapid immunoassays for diagnosis of heparin-induced thrombocytopenia: Comparison of diagnostic accuracy, reproducibility, and costs in clinical practice

**DOI:** 10.1371/journal.pone.0178289

**Published:** 2017-06-08

**Authors:** Andriyana Bankova, Yvonne Andres, Michael P. Horn, Lorenzo Alberio, Michael Nagler

**Affiliations:** 1 Division of Haematology and Central Haematology Laboratory, Luzerner Kantonsspital, Lucerne, Switzerland; 2 Kreisspital für das Freiamt, Muri, Switzerland; 3 University Institute of Clinical Chemistry, Inselspital, Bern University Hospital, University of Bern, Bern, Switzerland; 4 Centre of Laboratory Medicine, Inselspital, Bern University Hospital, University of Bern, Bern, Switzerland; 5 Division of Hematology and Central Hematology Laboratory, Centre Hospitalier Universitaire Vaudois, Lausanne, Switzerland; 6 University of Lausanne, Lausanne, Switzerland; 7 Department of Haematology, Inselspital, Bern University Hospital, University of Bern, Berne, Switzerland; 8 Department of Clinical Research, University of Bern, Berne, Switzerland; Institut d'Investigacions Biomediques de Barcelona, SPAIN

## Abstract

**Background:**

Immunoassays are crucial in the work-up of patients with suspected heparin-induced thrombocytopenia (HIT) and rapid tests have been recently developed. However, comparative data on diagnostic accuracy, reproducibility, and analytical costs of different immunoassays in clinical practice are limited.

**Methods:**

Samples of 179 consecutive patients evaluated for suspected HIT in clinical practice using a polyspecific enzyme-linked immunoabsorbent assay (GTI diagnostics; ELISA) and a rapid particle gel immunoassay (PaGIA), were additionally analysed with a IgG-specific chemiluminescent immunoassay (AcuStar HIT-IgG). Presence of HIT was defined as a positive functional heparin-induced platelet aggregation test. Diagnostic accuracy was determined for low, intermediate and high thresholds as previously established (ELISA: optical density 0.4, 1.3, and 2.0 respectively; PaGIA: positive/negative, titre of 4, titre of 32; AcuStar HIT-IgG: 1.0 U/ml, 2.8, 9.4) and reproducibility was assessed by repeated measurements. Costs of test determination were calculated taking reagents, controls, and working time of technicians according to Swiss health care system into account.

**Results:**

Data on PaGIA results were available for 171 patients (95.5%), ELISA for 144 patients (80.4%), and AcuStar HIT-IgG for 179 patients (100%). Sensitivity was above 95% for all assays at low and intermediate thresholds. Specificity increased with higher thresholds and was above 90% for all assays with intermediate and high thresholds. Specificity of AcuStar HIT-IgG (92.8%; 95% CI 87.7, 96.2) was significantly higher than PaGIA (83.0%; 95% CI 76.3, 88.5) and higher than ELISA (81.8%, 95% CI 74.2, 88.0) at low threshold (p<0.05). Reproducibility was adequate for all assays. Total costs per test were CHF 51.02 for ELISA, 117.70 for AcuStar HIT-IgG, and 83.13 for PaGIA.

**Conclusions:**

We observed favourable diagnostic accuracy measures and a high reproducibility for PaGIA and AcuStar HIT-IgG. Implementation into 24-hours-service might improve patient care but the results must be confirmed in other settings and larger populations as well.

## Introduction

The work-up of patients with suspected heparin-induced thrombocytopenia is difficult due to major practical problems and methodological restrictions [1]. Frequently, suspicion is expressed at times when haematological consultancy services and sophisticated laboratory tests are not immediately available: during night-shifts and weekends. However, a decision whether or not heparin shall be stopped and treatment with alternative anticoagulants started must be taken rapidly to prevent major complications [2]. Clinical scoring systems such as the 4Ts score are available to guide decision-making at bedside, but rating is difficult in inexperienced hands [1,3–5] and their positive predictive value is too low for diagnosing HIT [6,7]. Functional assays such as the serotonin release assay (SRA), the heparin-induced platelet activation assay (HIPA) or the heparin-induced platelet aggregation test (PAT) are regarded as gold-standard for the diagnosis of HIT. However, these tests are time-consuming and available in very few laboratories only [1]. Thus, physicians mostly rely on immunoassays to establish the presence of anti-platelet factor 4 (PF4)/heparin antibodies.

The diagnostic accuracy of enzyme-linked immunoassays (ELISA), which are used most frequently, is well evaluated [8]. However, ELISA results are available once daily at most because determination is time-consuming and requires a specialized laboratory [1]. Several rapid immunoassays were developed to overcome this limitation: particle gel immunoassay (PaGIA), lateral flow immunoassay, latex immunoturbidimetric assay, and chemiluminescent immunoassay. PaGIA and lateral flow immunoassay can be implemented in routine laboratories, because they do not require specialized analysers or expert knowledge. Chemiluminescent immunoassay and latex agglutination assay can be easily automated. However, evaluation studies comparing the diagnostic accuracy, reproducibility and costs of these assays in clinical practice are very limited, particularly with regard to the Swiss health care system [1,8,9].

To address these issues, we conducted a comprehensive evaluation study comparing accuracy, reproducibility, and costs of all commonly used immunoassays in clinical practice.

## Methods

### Study design and population

To estimate the utility of immunoassays for diagnosis of HIT in clinical practice, we selected a cohort of patients evaluated for suspected HIT at our institution (Inselspital University Hospital, University of Bern, Switzerland). To avoid any selection bias, we screened the database for a period with 190 consecutive patients with residual plasma samples. The following clinical data were extracted retrospectively: age, sex, setting (surgery, intensive care unit [ICU], or internal medicine), as well as results of the 4Ts score. We recorded quantitative results of ELISA, semi-quantitative results of PaGIA, and PAT if conducted at the time of assessment. We accepted missing test determinations, because we focused our investigation on real-life clinical practice. Residual citrated plasma samples were used to additionally perform a recently developed rapid immunoassay, the IgG-specific chemiluminescent immunoassay (AcuStar HIT-IgG). The study received approval by the local Ethics review board (Kantonale Ethikkommission Bern; 21.12.2014).

### Work-up of patients with suspected HIT in clinical practice

A protocol for management of patients with suspected HIT was implemented in 2004 and periodically renewed. Haematology consultancy team was informed by attending physicians or laboratory technicians (HIT test requested). Haematologist contacted attending physicians to clarify clinical probability, predominantly with the help of the 4Ts score [6]. PaGIA was conducted in most cases as a preliminary rapid test within few hours, followed by a polyspecific ELISA in the course of the following week. PAT was conducted as a confirmatory test every three months at the discretion of the haematology consultant.

### Handling of samples and determination of immunoassays

Blood samples were obtained using citrated plastic syringes (Monovette®, Sarstedt, Nümbrecht, Germany; 1:10 trisodium citrate 0.106 mol/L). Platelet poor plasma was generated by double centrifugation at 1500 g x 10 min. Aliquots were snap-frozen and stored at -70°C [7]. PaGIA (DiaMed SA, Cressier sur Morat, Switzerland) was conducted by pipetting 10 μL of patient plasma to the upper reaction chamber, followed by 50 μL of particle suspension. Results were recorded after 5 minutes of incubation at room temperature and 10 minutes’ centrifugation with the DiaMed ID-centrifuge. Semi-quantitative results were obtained by titration studies as previously described [10]. Polyspecific ELISA was determined as reported elsewhere using a microtiter plate reader, measured at 405 nm (GTI-PF4 Enhanced, Genetic Testing Institute, Waukesha, WI, USA [7]). AcuStar HIT-IgG (Instrumentation Laboratory, Bedford, MA, USA) was conducted in batches on a BIO-FLASH® analyser (Inova Diagnostics, San Diego, California, USA). Samples were thawed rapidly at 37°C. Assays were calibrated using AcuStar HIT-IgG calibrator 1+2, and controls were tested before every test run [11]. Results of 4Ts score, ELISA and PAT were not available to the technician conducting AcuStar HIT assay.

### Definition of HIT

HIT was defined as a positive functional assay, the PAT test. Following recent guidelines and recommendations, PAT was not conducted in a number of patients with a negative ELISA and/or a negative PaGIA in combination with a low risk/ intermediate risk 4Ts score [7]. These cases were classified as HIT negative. Inconclusive results were excluded as recommended by several authors (n = 8) [12]. We conducted two sensitivity analyses considering different definitions of HIT to address possible issues regarding PAT as the reference gold standard: (1) a broad definition of HIT was applied, considering patients with an ELISA result above OD 2.5 or an PaGIA titre of at least 32 in addition to PAT positive samples, and (2) considering PAT as definition of HIT only, excluding all patients without PAT results.

We determined a two-point PAT as previously described using a commercially available light transmission aggregometer APACT 4004 (LABiTec, Ahrensburg, Germany) [13,14]. Patient plasma was tested using platelet rich plasma from 4 selected donors. PAT was classified as positive if aggregation was more than 50% in at least 2 out of 4 donors with 0.5 U/mL heparin and suppressed with 100 U/mL heparin. Neither PaGIA nor AcuStar HIT-IgG results were available to the technician performing PAT and no other result were available to the technician conducting PaGIA.

### Costs

We calculated the analytical cost for determining the individual tests taking the following factors into account: reagents, controls, calibrators, consumables, and work expended. We assumed that 5 tests per week are determined, that tests are conducted on different days in case of PaGIA, AcuStar HIT-IgG, and that ELISA is run once weekly analysing all 5 samples in one run. In case of PaGIA, we applied 2.3 steps of titration per analysis (average number in our institution). Costs are based the on Swiss health care system. Labor costs were estimated according to the average wage of a qualified technician in Switzerland (CHF 42,- per hour).

### Statistical analysis

Descriptive statistics was used to characterise the study population. Accuracy was determined by calculating the sensitivity and specificity as well as likelihood ratios for the individual tests according to the presence of HIT. Calculations were repeated using two additional definitions of HIT as sensitivity analysis. Specificities between different thresholds and assays were compared using the z-test. In addition, post-test probabilities of different test results according to pre-test probability were reported. Calculation of diagnostic accuracy was done at three different thresholds; all of them are already established [1,7]. The low threshold corresponds to the recommendation of the manufacturer. The intermediate and high thresholds were identified in previous diagnostic accuracy studies with larger patient populations using receiver operating characteristics analysis [7,15–19]. Thus, an intermediate threshold of PaGIA does not directly correspond to an intermediate threshold of ELISA. Reproducibility was determined by calculating coefficients of variation (CV) for within-run repetitions (10 times) as well as day-to-day (5 times). A CV of 0.1 was set as requirement for imprecision, according to general principles of ELISA, clinical considerations and previous publications [20–23]. We calculated a sample size of 190 to demonstrate differences in specificity. We anticipated a frequency of true-negative values of 85%, considered a difference in specificity of 5% to be clinically relevant and a fixed a 95% CI. Analyses were performed using the Stata 13.1 statistics software package. (StataCorp. 2013. Stata Statistical Software: Release 13. College Station, TX: StataCorp LP), Figs were created using Prism 6 (GraphPad Software, Inc., La Jolla California USA).

## Results

### Patients

**Fig 1:**
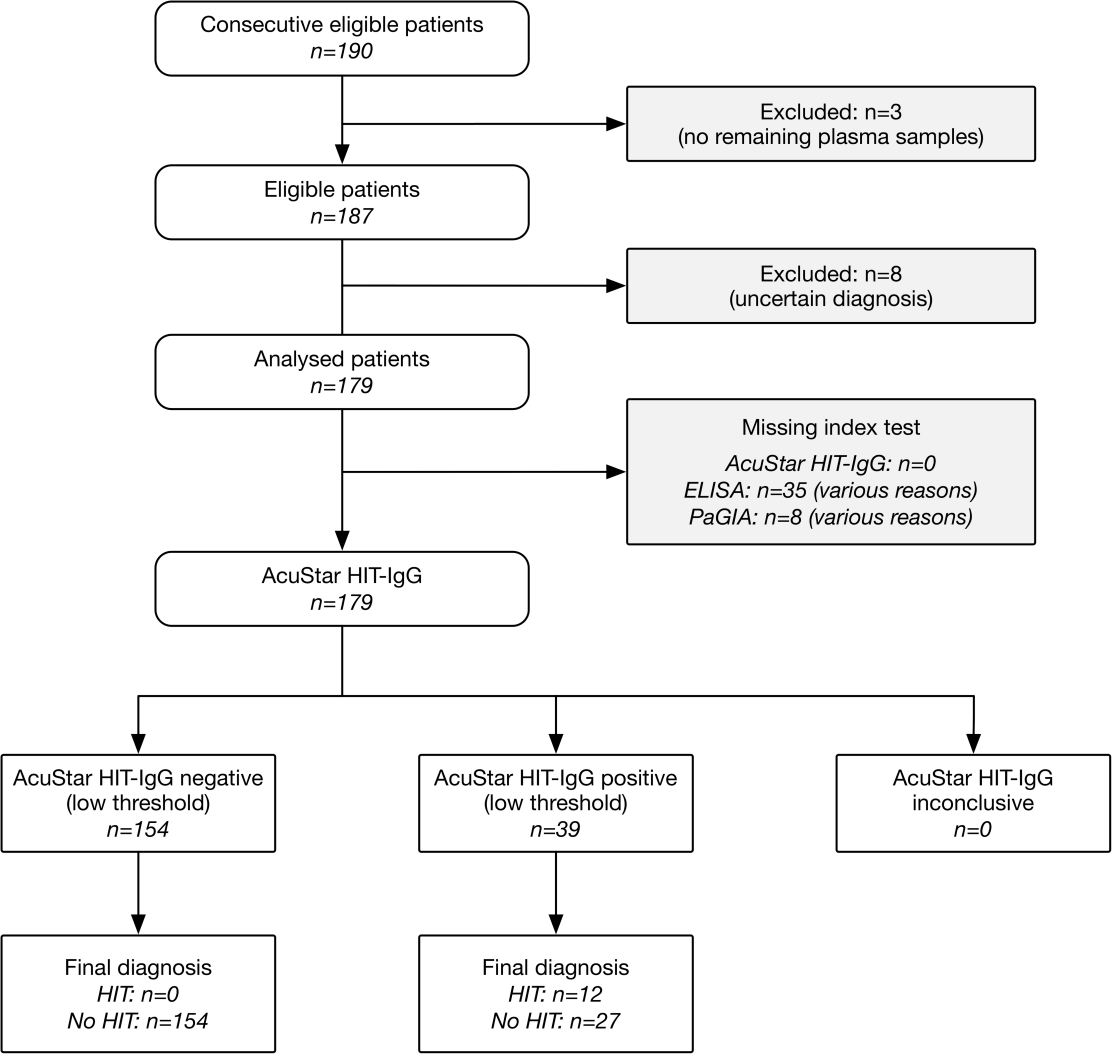
Flow of participants.

The flow of participants is shown in Fig 1. Residual plasma samples were available in 187 out of 190 patients. Eight patients were excluded from analysis because of uncertain diagnosis of HIT. Finally, 179 patients were included for analysis. Among them, AcuStar HIT-IgG was conducted in 179 patients (100%), PaGIA in 171 patients (95.5%), and ELISA in 144 patients (80.4%). Median age was 70.0 years (inter-quartile range [IQR] 61.3, 76.9), 42.5% were female. Detailed patient characteristics are shown in Table 1. The clinical setting was surgery in 38.6% of the cases, internal medicine in 32.4% of the patients, and intensive care unit in 29.1% of the patients. The clinical risk according to 4Ts score was estimated to be low in 24.6%, intermediate in 15.6%, and high in 1.7%. The 4Ts score was missing in 104 patients (58.1%) because samples were received from external hospitals.

**Table 1:** Patient characteristics according to the presence of HIT.

Characteristics	HIT negative	HIT positive	All samples
	*Numbers (percent)*		
**Patients**	166 (92.7)	13 (7.3)	179 (100)
**Age** *Median (IQR)*	70.5 (61.4, 80.0)*	70.9 (65.5, 80.8)^+^	70.0 (61.3, 76.9)^#^
**Female sex**	74 (44.6)	2 (15.4)	76 (42.5)
**Setting**			
Surgery	61 (36.8)	8 (61.5)	69 (38.6)
ICU	49 (29.5)	3 (23.1)	52 (29.1)
Internal medicine	56 (33.7)	2 (15.4)	58 (32.4)
**4T’s score**			
Low risk	44 (26.5)	0	44 (24.6)
Intermediate risk	22 (13.3)	6 (46.2)	28 (15.6)
High risk	2 (1.2)	1 (7.7)	3 (1.7)
Missing score	98 (59.0)	6 (46.2)	104 (58.1)

* range 22.4, 94.5

^+^ range 39.6, 82.4

^#^ range 22.4, 94.5

### Antibody test results

**Fig 2:**
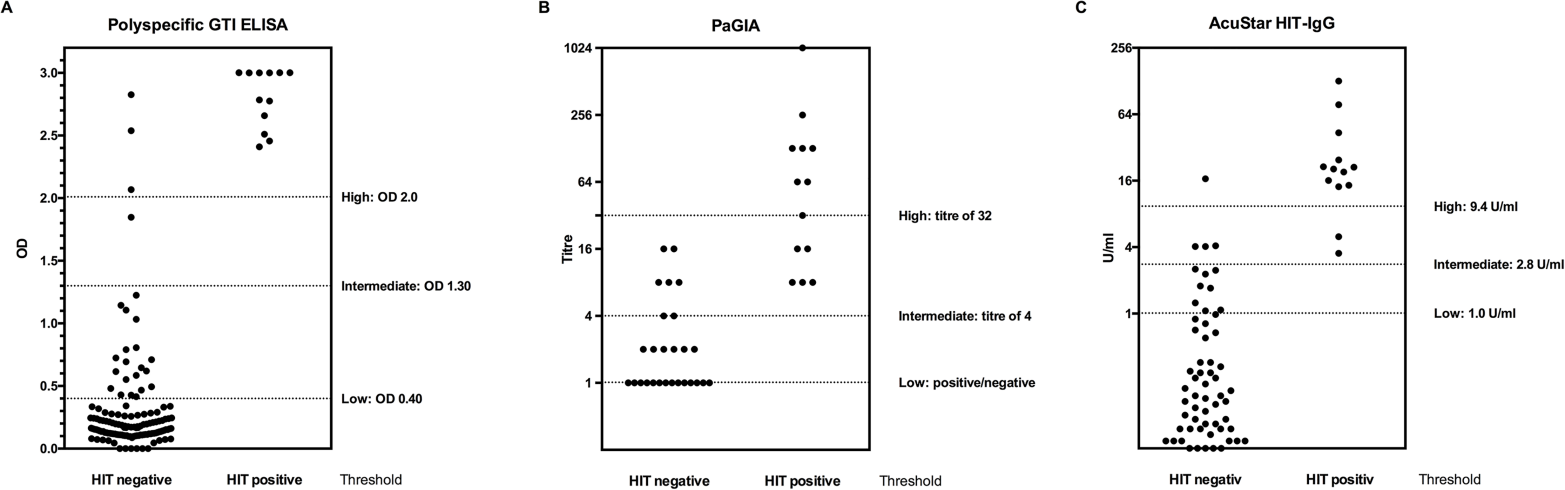
Accuracy of different immunoassays for diagnosis of HIT in clinical practice. Distribution of quantitative results according to presence of HIT (positive or negative heparin-induced platelet aggregation test [PAT] respectively and clinical criteria) is shown for different thresholds of the test. (A) enzyme-linked immunoassay (ELISA), (B) particle gel immunoassay (PaGIA), and (C) IgG-specific chemiluminescent immunoassay (AcuStar HIT-IgG).

Thirteen patients were classified as HIT-positive because of a positive PAT test. One hundred and forty-one patients were classified as HIT-negative because of a negative PAT, and 21 patients because of a negative ELISA and/or PaGIA result and a low/intermediate 4Ts score. Thus, the prevalence of HIT in the present study population was 7%. ELISA was conducted in 144 patients (80.0%), with a major difference between HIT-positive patients (median OD 2.90) and HIT-negative patients (median OD 0.17; Table 2; Fig 2). Results were missing in 41 patients (22.9%), mostly because of a negative PaGIA and a low risk 4Ts score. Data on PaGIA results were available for 171 patients (95.5%), the distribution among HIT-positive and HIT-negative patients is shown in Table 2 and Fig 2. AcuStar HIT-IgG were conducted in all 179 samples resulting in very low levels in HIT-negative patients and high levels in HIT-positive samples (Table 2; Fig 2).

**Table 2:** Immunoassay results according to the presence of HIT.

Immunoassay			HIT negative	HIT positive	Available observations
				*Median (IQR)*	*Numbers (Percent)*
PaGIA		*Titre*	0 (0, 0)	64 (16, 128)	172 (96.1)
Polyspecific ELISA (GTI)		*OD*	0.17 (0.11, 0.28)	2.90 (2.58, 3.00)	144 (80.4)
AcuStar HIT-IgG		*U/ml*	0.02 (0.00, 0.10)	20.44 (14.55, 24.71)	179 (100)

### Comparative diagnostic accuracy

Quantitative immunoassay results were considerably higher in patients with HIT compared to patients without HIT in all of the tests (Table 2). The distribution of results in relation to different thresholds of the immunoassays is shown in Fig 2. In the current population, sensitivity was 100% for all assays at low and intermediate thresholds (Table 3). However, the 95% confidence intervals are wide. At high threshold, sensitivity was 84.6% for the AcuStar HIT-IgG and 58.3% for PaGIA only. Specificity was considerably below 90% at low thresholds for all assays except for AcuStar HIT-IgG (92.8%; 95% CI 87.7, 96.2). Specificity increased significantly at intermediate thresholds (PaGIA: 95.6%; 95%CI 91.1, 98.2; ELISA: 96.8; 92.4, 99.2; AcuStar HIT-IgG: 97.6; 94.0, 99.3). Specificity of AcuStar HIT-IgG was significantly higher than PaGIA and ELISA at low threshold.

**Table 3:** Diagnostic accuracy of immunoassays according to PAT result.

Immunoassay	TP	FN	FP	TN	Sensitivity	Specificity	Difference in specificity	Likelihood ratio	
					*Percent* *(95%CI)*		*p<0*.*05* *compared to*	*Positive* *(95%CI)*	*Negative* *(95%CI)*
**PaGIA**									
Low threshold *(Positive/ negative)*	12	0	27	132	100.0 (73.5, 100.0)	83.0 (76.3, 88.5))	AcuStar HIT-IgG	5.9 (4.2, 8.3)	0.0
Intermediate threshold *(Titre of 4)*	12	0	7	152	100.0 (73.5, 100.0)	95.6^¶^ (91.1, 98.2)		22.7 (11.0, 46.9)	0.0
High threshold *(Titre of 32)*	12	5	0	159	58.3 (27.7, 84.8)	100.0^#^ (97.7, 100.0)		>1000	0.29
**Polyspecific ELISA (GTI)**									
Low threshold *(OD 0*.*4)*	12	0	24	108	100.0 (73.5, 100.0)	81.8 (74.2, 88.0)	AcuStar HIT-IgG	5.5 (3.8, 7.9)	0.0
Intermediate threshold *(OD 1*.*3)*	12	0	4	128	100.0 (73.5, 100.0)	96.8^¶^ (92.4, 99.2)		33.0 (12.6, 86.6)	0.0
High threshold *(OD 2*.*0)*	12	0	3	132	100.0 (73.5, 100.0)	97.7 (93.5, 99.5)		44.0 (14.4, 130.0)	0.0
**AcuStar HIT-IgG**									
Low threshold *(1*.*0 U/ml)*	13	0	12	154	100.0 (75.3, 100.0)	92.8 (87.7, 96.2)	PaGIA, ELISA	13.8 (8.0, 23.9)	0.0
Intermediate threshold *(2*.*8 U/ml)*	13	0	4	162	100.0 (75.3, 100.0)	97.6^¶^ (94.0, 99.3)		41.5 (15.8, 110.0)	0.0
High threshold *(9*.*4 U/ml)*	11	2	1	165	84.6 (54.6, 98.1)	99.4 (96.7, 100.0)		140.5 (19.6, 1000.0)	0.15 (0.04, 0.55)

TP, true positives; FN, false negatives; FP, false positives; TN, true negatives

¶ p<0.05 for difference in specificity compared to low threshold

# p<0.05 for difference in specificity compared to intermediate threshold

Essentially identical results were observed in first sensitivity analysis, considering PAT as reference gold standard only (Table A and Table B in S1 File). Sensitivity analysis 2, considering a broad definition of HIT revealed lower numbers for sensitivity and a comparable specificity for all of the assays. However, sensitivity was above 90% at low thresholds as well.

### Reproducibility

Within-run imprecision was within predefined limits for all assays (CV<0.1). A CV of 0.04 was determined for ELISA, and 0.03 for AcuStar HIT-IgG. Antibody titre was identical in 9 out of 10 PaGIA measurements (one replicate with a titre of 4 instead of 2).

Day-to-day variation was within the predefined limits as well (CV≤0.1). For ELISA, CV was 0.07, and for AcuStar IgG 0.03. Four out of 5 PaGIA titres were identical (a titre of 4 instead of 2 was seen in one sample) confirming previous results [22].

### Costs

We calculated total costs per test for the individual assays as follows: CHF 51.02 for ELISA, CHF 117.70 for AcuStar HIT-IgG, and 83.13 for PaGIA. In case of ELISA, reagents summed up to CHF 26.10 per sample (including controls and calibrators), consumables to CHF 1.90, and average wage of CHF 24.5 (175 minutes of work expended). For PaGIA, reagents costs CHF 65.19, consumables 0.44, and wage CHF 17.50 (25 minutes). Costs of AcuStar HIT-IgG were CHF 106.20 for reagents, CHF 1.00 for consumables, and CHF 10.50 for wages (15 minutes).

## Discussion

As observed in clinical practice, all immunoassays demonstrated adequate diagnostic accuracy measures at intermediate thresholds; sensitivity was above 95% and specificity above 90%. In general, specificity was higher with AcuStar HIT-IgG and lower at low thresholds for all assays. A good reproducibility was shown for all tests. Relevant differences exist with regard to analytical costs in the Swiss health care system.

Even though this study was conducted in clinical practice, our results are in-line with previous publications. First polyspecific ELISA were developed in the nineties, and a number of studies evaluated their diagnostic accuracy [8]. A recent meta-analysis estimated the pooled sensitivity to be 96.7% at low threshold and 98.4% at intermediate threshold [8] (specificity 86.8% and 94.9% respectively). In the same meta-analysis, diagnostic accuracy of PaGIA was calculated to be 96.5% (low threshold), and 98.9% (intermediate threshold). Specificity of PaGIA was 93.7% and 95.9% respectively. Specificity of PaGIA was considerably lower in our investigation, probably due to a limited precision (restricted number of patients) or some inconsistencies of test determination in clinical practice. Fewer studies focused on chemiluminescent immunoassay [11,15,16,24–26], pooled data are available from the same meta-analysis (sensitivity at low/ intermediate thresholds for AcuStar HIT-IgG 98.9%/ 78.6%; specificity of AcuStar HIT-IgG 94.9%/ 98.7%). In our data, sensitivity was higher at intermediate thresholds, most likely because of a limited number of HIT-positive patients.

Most previous data on reproducibility originate from a North American proficiency testing program [23]. CV was 0.29 for a polyspecific ELISA (GTI). Our data, showing much lower variation, suggest relevant variation in laboratory procedures as a source for this variability. A good reproducibility was shown in one study for PaGIA [10,22] and in another for chemiluminescent immunoassay as well [11].

Costs of “rapid” tests, PaGIA and chemiluminescent immunoassays were higher compared to the ELISA assay. However, rapid immunoassays can be conducted in a 24 hours service, reducing the time span between suspicion of HIT and antibody testing from several days to few hours. A recent published budget model estimated that this might save a relevant amount of anticoagulant treatment costs, by far outweighing the higher analytical cost [9].

The strengths of our study are that we included consecutive patients evaluated for HIT in clinical practice. Thus, our results are applicable to real-life practice. In addition, we evaluated a number of important criteria that determine the utility in clinical practice in a joint assessment: accuracy, reproducibility, as well as costs. Furthermore, we directly compared commonly used immunoassays. Even more, we applied the costs of the Swiss health care system, providing recommendations for Swiss physicians and laboratory managers in particular.

Our study has several important limitations. First, the number of observations and the number of true HIT cases is limited. This is reflected by wide confidence intervals and a sensitivity of 100%, which is higher than previously estimated [8]. However, a high sensitivity of all assays is already known and our primary outcome was specificity. In addition, our results obtained in clinical practice confirm previous studies conducted in more rigorous settings and this is the most important message of the present investigation [8,27]. Second, a relevant number of measurements are missing and some patients had to be excluded due to missing plasma samples (n = 3) or PAT results (n = 8). This might lead to selection of patients and affect the internal validity of the study. However, we focused on a high applicability to clinical practice, which is an important issue in diagnostic accuracy studies as well [28]. In addition, exclusion of patients with inconclusive reference standard results is recommended by other authors [12]. Third, PAT as reference standard is criticized because of a limited sensitivity [1]. We conducted a sensitivity analysis to address this issue by applying a broad definition of HIT, considering all patients as HIT positive with an ELISA OD above 2.0 and/ or an PaGIA titre of at least 8 (Table C and D of S1 File). Sensitivity was more limited for all assays but still similar to previous publications [8] with a comparable pattern between assays and thresholds. Finally, our analysis of cost was based on the Swiss health care system limiting the applicability to different countries. Moreover, we did not take the analyzer for determination of chemiluminescent immunoassay into account, assuming that this test will only be considered if a respective analyzer by Instrumentation Laboratory, is already implemented for other purposes. Another limitation is that we conducted chemiluminescent immunoassays retrospectively on stored samples. Thus, our investigation might not reflect inconsistencies of test determination in real-life practice.

Our data suggest that rapid immunoassays have accuracy and reproducibility properties that are at least as good as an ELISA assay. Furthermore, they have the potential to relevantly improve patient care due to a shorter turn-around time. Not only that unnecessary switching to alternative anticoagulants will be avoided, but the risk of thromboembolic complications while waiting for laboratory results will be prevented. Even more, they offer the potential of relevantly saving treatment-related health care costs [9]. The easy handling of PaGIA without the need for specialized knowledge or analyzers facilitates the implementation of a 24-hours-service even in small to middle-sized medical laboratories. In contrast, chemiluminescent immunoassays are fully automatable. We suggest applying intermediate thresholds at least for polyspecific assays in order to improve specificity. However, we do not recommend to implement an intermediate threshold for AcuStar HIT-IgG because sensitivity was limited in other studies [8].

Our study was restricted on the presence of HIT as the only clinical outcome. Future prospective multicenter studies might focus on clinical outcomes such as thromboembolic, bleeding complications, and deaths as well. In addition, evaluation studies are needed in order to investigate the diagnostic accuracy and clinical outcomes of complete diagnostic algorithms comprising clinical scoring systems immunoassays as well.

In conclusion, rapid immunoassays for diagnosis of HIT have favorable characteristics in terms of diagnostic accuracy and reproducibility in clinical practice. The implementation of 24-hour service using these tests might not only improve care in patients with suspected HIT but has the potential to save treatment-related health care costs.

## Supporting information

S1 File Table A: Sensitivity analysis—Patient characteristics according according to PAT assay; Table B: Sensitivity analysis—Diagnostic accuracy of immunoassays according to PAT result; Table C: Sensitivity analysis—Patient characteristics according to a broad definition of HIT; Table D: Sensitivity analysis—Diagnostic accuracy of immunoassays according to a broad definition of HIT; Table E: STARD Checklist.
(DOCX)
