# Supplementary material for: Rapid immunoassays for diagnosis of heparin-induced thrombocytopenia: Comparison of diagnostic accuracy, reproducibility, and costs in clinical practice
Source: PLoS One. 2017 Jun 8;12(6):e0178289. doi: 10.1371/journal.pone.0178289 (PMC5464550; doi:10.1371/journal.pone.0178289)
Supplement: S1 File — Table A: Sensitivity analysis—Patient characteristics according according to PAT assay; Table B: Sensitivity analysis—Diagnostic accuracy of immunoassays according to PAT result; Table C: Sensitivity analysis—Patient characteristics according to a broad definition of HIT; Table D: Sensitivity analysis—Diagnostic accuracy of immunoassays according to a broad definition of HIT; Table E: STARD Checklist. (DOCX) [file pone.0178289.s001.docx]

**Table A: Sensitivity analysis - Patient characteristics according according to PAT assay**

| **Characteristics** | **PAT negative** | **PAT positive** | **PAT not determined** | **All samples** |
| --- | --- | --- | --- | --- |
|  | *Numbers (percent)* | | | |
| **Patients** | 145 (77.5) | 13 (7.0) | 29 (15.5) | 187 (100) |
| **Age** *Median (IQR)* | 70.8 (60.7, 77.7) | 70.9 (65.5, 80.8) | 67.0 (58.9, 70.8) | 70.0 (61.3, 76.9) |
| **Female sex** | 62 (42.8) | 2 (15.4) | 13 (44.8) | 77 (41.2) |
| **Setting** |  |  |  |  |
| Surgery | 57 (39.3) | 8 (61.5) | 7 (24.1) | 72 (38.5) |
| ICU | 40 (27.6) | 3 (23.1) | 12 (41.4) | 55 (29.4) |
| Internal medicine | 48 (33.1) | 2 (15.4) | 10 (34.5) | 60 (32.1) |
| **4T’s score** |  |  |  |  |
| Low risk | 43 (29.7) | 0 | 3 (10.3) | 46 (24.6) |
| Intermediate risk | 19 (13.1) | 6 (46.2) | 3 (10.3) | 28 (15.0) |
| High risk | 2 (1.4) | 1 (7.7) | 0 | 3 (1.6) |
| Missing score | 81 (55.9) | 6 (46.1) | 23 (79.4) | 110 (58.8) |

**Table B: Sensitivity analysis - Diagnostic accuracy of immunoassays according to PAT result**

| **Immunoassay** | **Sensitivity** | **Specificity** | **Likelihood ratio** | |
| --- | --- | --- | --- | --- |
|  | *Percent*  *(95%CI)* | | *Positive*  *(95%CI)* | *Negative*  *(95%CI)* |
| **PaGIA** |  |  |  |  |
| Low threshold  *(Positive/ negative)* | 100.0  (69.9, 99.2) | 81.3  (73.7, 87.1) | 5.3  (3.8, 7.5) | 0.0 |
| Intermediate threshold  *(Titer of 4)* | 100.0  (73.5, 100.0) | 95.1  (90.2, 98.0) | 20.6  (10.0, 42.4) | 0.0 |
| High threshold  *(Titer of 32)* | 58.3  (27.7, 84.8) | 100.0  (97.5, 100.0) | >1000 | 0.05  (0.02, 0.11) |
| **Polyspecific ELISA (GTI)** |  |  |  |  |
| Low threshold  *(OD 0.4)* | 100.0  (69.9, 99.2) | 81.0  (72.8, 87.2) | 5.3  (3.7, 7.5) | 0.0 |
| Intermediate threshold  *(OD 1.3)* | 100.0  (69.9, 99.2) | 96.8^¶^  (91.6, 99.0) | 31.5  (12.0, 82.6) | 0.0 |
| High threshold  *(OD 2.0)* | 100.0  (69.9, 99.2) | 97.6  (92.7, 99.4) | 42.0  (13.7, 130.0) | 0.0 |
| **AcuStar HIT-Ab (polyspecific)** |  |  |  |  |
| Low threshold  *(1.0 U/ml)* | 100.0  (39.6, 97.5) | 77.1  (62.3, 87.5) | 4.4  (2.6, 7.3) | 0.0 |
| Intermediate threshold  *(2.8 U/ml)* | 100.0  (39.6, 97.6) | 91.7  (79.1, 97.3) | 12.0  (4.7, 30.7) | 0.0 |
| High threshold  *(9.4 U/ml)* | 100.0  (39.6, 97.6) | 97.9  (87.5, 99.9) | 48.0  (6.9, 300.3) | 0.0 |
| **AcuStar HIT-IgG** |  |  |  |  |
| Low threshold  *(1.0 U/ml)* | 100.0  (71.7, 99.3) | 91.7  (85.7, 95.5) | 12.1  (7.0, 20.8) | 0.0 |
| Intermediate threshold  *(2.8 U/ml)* | 100.0  (71.7, 99.3) | 97.2^¶^  (92.6, 99.1) | 36.3  (13.8, 95.3) | 0.0 |
| High threshold  *(9.4 U/ml)* | 84.6  (53.7, 97.3) | 99.3  (95.6, 100.0) | 122.7  (17.2, 800.8) | 0.15  (0.04, 0.55) |

**Table C: Sensitivity analysis - Patient characteristics according to a broad definition of HIT**

| **Characteristics** | **HIT negative** | **HIT positive** | **All samples** |
| --- | --- | --- | --- |
|  |  | *Numbers (percent)* |  |
| **Patients** | 162 (90.1) | 18 (9.9) | 180 (100) |
| **Age** *Median (IQR)* | 70.3 (61.3, 76.8) | 70.3 (65.5, 80.8) | 70.3 (61.6, 77.4) |
| **Female sex** | 72 (43.9) | 4 (22.2) | 76 (41.1) |
| **Setting** |  |  |  |
| Surgery | 60 (37.2) | 9 (50.0) | 70 (38.5) |
| ICU | 48 (29.3) | 6 (33.3) | 54 (29.7) |
| Internal medicine | 55 (33.5) | 3 (16.7) | 58 (31.8) |
| **4T’s score** |  |  |  |
| Low risk | 44 (26.8) | 0 | 44 (24.2) |
| Intermediate risk | 22 (13.4) | 6 (33.3) | 28 (15.3) |
| High risk | 2 (1.2) | 1 (5.6) | 3 (1.7) |
| Missing score | 96 (58.5) | 11 (61.1) | 107 (58.8) |

**Table D: Sensitivity analysis - Diagnostic accuracy of immunoassays according to a broad definition of HIT**

| **Immunoassay** | **Sensitivity** | **Specificity** | **Likelihood ratio** | |
| --- | --- | --- | --- | --- |
|  | *Percent*  *(95%CI)* | | *Positive*  *(95%CI)* | *Negative*  *(95%CI)* |
| **PaGIA** |  |  |  |  |
| Low threshold  *(Positive/ negative)* | 93.8  (69.8, 99.8) | 83.4  (76.7, 88.9) | 5.7  (3.9, 8.2) | 0.07  (0.01, 0.5) |
| Intermediate threshold  *(Titer of 4)* | 93.8  (69.8, 99.8) | 96.2  (91.9, 98.6) | 24.5  (11.1, 54.3) | 0.06  (0.01, 0.43) |
| High threshold  *(Titer of 32)* | 91.3  (72.0, 98.9) | 98.7  (95.4, 99.8) | >1000 | 0.44  (0.25, 0.76) |
| **Polyspecific ELISA (GTI)*** |  |  |  |  |
| Low threshold  *(OD 0.4)* | 100.0  (78.2, 100.0) | 83.1  (75.5, 89.1) | 5.9  (4.0, 8.6) | 0.0 |
| Intermediate threshold  *(OD 1.3)* | 100.0  (78.2, 100.0) | 98.5  (94.6, 99.8) | 65.0  (16.4, 260.0) | 0.0 |
| High threshold  *(OD 2.0)* | 100.0  (78.2, 100.0) | 99.2  (95.8, 100.0) | 130.0  (18.5, 920.0) | 0.0 |
| **AcuStar HIT-IgG** |  |  |  |  |
| Low threshold  *(1.0 U/ml)* | 100.0  (81.5, 100.0) | 93.9  (89.1, 97.0) | 16.4  (9.0, 29.9) | 0.0 |
| Intermediate threshold  *(2.8 U/ml)* | 94.4  (72.7, 99.9) | 98.2  (94.8, 99.6) | 51.6  (16.7, 160.0) | 0.06  (0.01, 0.38) |
| High threshold  *(9.4 U/ml)* | 72.2  (46.5, 90.3) | 100.0  (97.8, 100.0) | >1000 | 0.27  (0.13, 0.59) |

* Diagnostic accuracy measures of ELISA assay are not meaningful in this sensitivity analysis because diagnosis of HIT largely depends on ELISA results

**Table E: STARD Checklist**

|  | **Section & Topic** | **No** | **Item** | **Reported on page #** |
| --- | --- | --- | --- | --- |
|  |  |  |  |  |
|  | **TITLE OR ABSTRACT** |  |  |  |
|  |  | **1** | Identification as a study of diagnostic accuracy using at least one measure of accuracy  (such as sensitivity, specificity, predictive values, or AUC) | 1 |
|  | **ABSTRACT** |  |  |  |
|  |  | **2** | Structured summary of study design, methods, results, and conclusions  (for specific guidance, see STARD for Abstracts) | 2 |
|  | **INTRODUCTION** |  |  |  |
|  |  | **3** | Scientific and clinical background, including the intended use and clinical role of the index test | 4 |
|  |  | **4** | Study objectives and hypotheses | 5 |
|  | **METHODS** |  |  |  |
|  | *Study design* | **5** | Whether data collection was planned before the index test and reference standard  were performed (prospective study) or after (retrospective study) | 5 |
|  | *Participants* | **6** | Eligibility criteria | 5 |
|  |  | **7** | On what basis potentially eligible participants were identified  (such as symptoms, results from previous tests, inclusion in registry) | 5 |
|  |  | **8** | Where and when potentially eligible participants were identified (setting, location and dates) | 5 |
|  |  | **9** | Whether participants formed a consecutive, random or convenience series | 5 |
|  | *Test methods* | **10a** | Index test, in sufficient detail to allow replication | 6 |
|  |  | **10b** | Reference standard, in sufficient detail to allow replication | 6/7 |
|  |  | **11** | Rationale for choosing the reference standard (if alternatives exist) | 6/7 |
|  |  | **12a** | Definition of and rationale for test positivity cut-offs or result categories  of the index test, distinguishing pre-specified from exploratory | 7 |
|  |  | **12b** | Definition of and rationale for test positivity cut-offs or result categories  of the reference standard, distinguishing pre-specified from exploratory | 7 |
|  |  | **13a** | Whether clinical information and reference standard results were available  to the performers/readers of the index test | 6 |
|  |  | **13b** | Whether clinical information and index test results were available  to the assessors of the reference standard | 7 |
|  | *Analysis* | **14** | Methods for estimating or comparing measures of diagnostic accuracy | 7/8 |
|  |  | **15** | How indeterminate index test or reference standard results were handled | 8-10 |
|  |  | **16** | How missing data on the index test and reference standard were handled | 8-10 |
|  |  | **17** | Any analyses of variability in diagnostic accuracy, distinguishing pre-specified from exploratory | N/A |
|  |  | **18** | Intended sample size and how it was determined | 8 |
|  | **RESULTS** |  |  |  |
|  | *Participants* | **19** | Flow of participants, using a diagram | 9 |
|  |  | **20** | Baseline demographic and clinical characteristics of participants | 8 |
|  |  | **21a** | Distribution of severity of disease in those with the target condition | 9 |
|  |  | **21b** | Distribution of alternative diagnoses in those without the target condition | 9 |
|  |  | **22** | Time interval and any clinical interventions between index test and reference standard | 6 |
|  | *Test results* | **23** | Cross tabulation of the index test results (or their distribution)  by the results of the reference standard | 10 |
|  |  | **24** | Estimates of diagnostic accuracy and their precision (such as 95% confidence intervals) | 10 |
|  |  | **25** | Any adverse events from performing the index test or the reference standard | N/A |
|  | **DISCUSSION** |  |  |  |
|  |  | **26** | Study limitations, including sources of potential bias, statistical uncertainty, and generalisability | 12/13 |
|  |  | **27** | Implications for practice, including the intended use and clinical role of the index test | 13/14 |
|  | **OTHER INFORMATION** |  |  |  |
|  |  | **28** | Registration number and name of registry | 5 |
|  |  | **29** | Where the full study protocol can be accessed | N/A |
|  |  | **30** | Sources of funding and other support; role of funders | N/A |
|  |  |  |  |  |
